# A TCM Formula YYWY Inhibits Tumor Growth in Non-Small Cell Lung Cancer and Enhances Immune-Response Through Facilitating the Maturation of Dendritic Cells

**DOI:** 10.3389/fphar.2020.00798

**Published:** 2020-06-09

**Authors:** Bei Zhao, Xiaodan Hui, Lijing Jiao, Ling Bi, Lei Wang, Piao Huang, Wenxiao Yang, Yinan Yin, Shenyi Jin, Chengyan Wang, Xue Zhang, Ling Xu

**Affiliations:** ^1^Department of Oncology, Yueyang Hospital of Integrated Traditional Chinese and Western Medicine, Shanghai University of Traditional Chinese Medicine, Shanghai, China; ^2^Department of Wine, Food and Molecular Biosciences, Faculty of Agriculture & Life Sciences, Lincoln University, Christchurch, New Zealand; ^3^Institute of Clinical Immunology, Yueyang Hospital of Integrated Traditional Chinese and Western Medicine, Shanghai University of Traditional Chinese Medicine, Shanghai, China; ^4^A Center for Chemical Biology, Institute of Interdisciplinary Integrative Medicine Research, Shanghai University of Traditional Chinese Medicine, Shanghai, China

**Keywords:** non-small cell lung cancer, Traditional Chinese Medicine (TCM) formula, immunotherapy, dendritic cells, mitogen-activated protein kinase, NF-κB

## Abstract

In worldwide, lung cancer has a major socio-economic impact and is one of the most common causes of cancer-related deaths. Current therapies for lung cancer are still quite unsatisfactory, urging for alternative new treatments. Traditional Chinese Medicine (TCM) is currently increasingly popular and exhibits a complicated intervention in cancers therapy. In this study, we evaluated the anti-tumor effect and explored the mechanisms of a TCM formula Yangyinwenyang (YYWY) in non-small cell lung cancer (NSCLC) models. YYWY induced the apoptosis of lung cancer cells *in vitro*. In Lewis NSCLC-bearing mice model, YYWY significantly inhibited the tumor growth. Further, RNA-seq analysis and immunostaining of the tumor tissue implied the critical role of YYWY in the regulation of immune response, especially the dendritic cells (DCs) in the effect of YYWY. Therefore, we focused on DCs, which were the initiator and modulator of the immune response. YYWY facilitated the maturation of DCs through MAPK and NF-κB signaling pathways and promoted the release of the cytokines IFN-γ, interleukin (IL)-1β, IL-2, IL-12, and tumor necrosis factor (TNF)-α by DCs. Moreover, the YYWY-matured DCs enhanced the proliferation of T cells and promoted the differentiation of T cells into T helper Th1 and cytotoxic T cell (CTL). In addition, YYWY increased the ratio of Th1/Th2 (IFN-γ/IL-4 radio). Collectively, our findings clearly suggested that YYWY exerted an anti-tumor effect on NSCLC, at least partially through facilitating the mature DCs to activate the proliferation and differentiation of T cells.

## Introduction

Lung cancer is considered to be the leading cause of cancer death worldwide, with around 80–85% grouped as non-small cell lung cancer (NSCLC). Although targeted therapy to NSCLC driven by EGFR-activating mutations (Robichaux et al., 2018; Ahn et al., 2019) or ALK and ROS1 fusions has achieved marked survival advantages in the past decades, acquired resistance usually develops within 9–12 months in almost patients (Engle and Kolesar, 2014; Aguilar et al., 2019; Horn et al., 2019). Moreover, the median overall survival is only about 7.9 months in oncogene-negative patients with cytotoxic chemotherapy treatment (Paz-Ares et al., 2017). These existing therapies for NSCLC are still quite unsatisfactory to improve outcomes for lung cancer patients. Therefore, it is important for medical workers to explore new therapeutics and drugs (Desantis et al., 2014).

Immunotherapy has made great progression owing to the constantly understanding and enriched knowledge of cancer immunology, altering the therapeutic landscape of pan-negative advanced NSCLC dramatically. Progress of the cancer-immunity cycle (Daniel and Ira, 2017) requires the presence of activation signals that allow dendritic cells (DCs) to mature, migrate to the lymph nodes and present the neo-antigens to naïve T cells. Afterwhile, mature DCs with suitable signals are able to active T cell effector functions (Evelyn et al., 2019), which target and kill cancer cells (Gebhardt et al., 2018; Flood et al., 2019; Miao et al., 2019; Nobuhiro et al., 2019). Hence, strategies to initiate or enhance cancer immunity may achieve a promising result in cancer immunotherapy.

As the initial antigen-presenting cell in immune response (Coquerelle and Moser, 2010), after exposuring to pathogenic or inflammatory molecules, DCs mature and possess stronger antigen presentation and immunostimulatory capacity to activate T cells and help to guide T cell differentiation by up-regulating the major histocompatibility complex (MHC), co-stimulating molecules (such as CD40, CD80, CD83, and MHCII) and increasing secreted cytokines like tumor necrosis factor (TNF)-α, interleukin (IL)-12, IL-2, and IL-1β (Banchereau and Steinman, 1998; Ardavín et al., 2004; Mallick et al., 2009). Since DCs play a crucial role in modulating immune responses, it is considered promising for intervention. It is worth mentioning that the efficacy of DCs against cancer was confirmed in clinical trials which was supported by that antigen-loaded DCs induced the anti-tumor immunological effect in cancer patients (Wilgenhof et al., 2016; Cheong and Sun, 2018).

Traditional Chinese medicine (TCM) has attracted caused extensive concern in cancer therapies (Liao et al., 2017; Chen et al., 2019; Liu Y.-X. et al., 2019) due to its synergistic effects and low side effects. Jinfukang (JFK), a Chinese herbal formula consisted of 12 herbs, has been used clinically for the treatment of NSCLC in China for decades (Lu et al., 2016; Zujun et al., 2019). Our previous studies indicated that JFK induced cellular apoptosis through activation of AIFM2, Fas, and DR4. In addition, it has been reported that TCM could exert synergistic effects in combination with chemotherapy on lung cancer cell apoptosis (Lu et al., 2016; Lu et al., 2018). According to the further computational algorithms, our research group developed the optimized formula Yangyinwenyang (YYWY) based on JFK, which consists of *Paris polyphylla*, *Gynostemma pentaphyllum*, *Liriope graminifolia*, and *Fenugreek*. However, the anti-lung cancer effect of YYWY and the underlying mechanisms still need to investigate. In this study, we reported that YYWY significantly induced the apoptosis of lung cancer cells and inhibited tumor growth in Lewis-bearing lung cancer mice. Further, RNA-seq results suggested DCs played a critical role in the anti-lung cancer effect of YYWY. We found that YYWY promoted the maturation of DCs, which induced the proliferation and differentiation of T cells into T helper 1 (Th1) and cytotoxic T cell (CTL) through regulating the MAPK and NF-κB signaling pathways. Collectively, our study suggested YYWY is an effective candidate of anti-NSCLC through immunoregulation.

## Materials and methods

### Preparation of YYWY

The raw herbs were provided by Huayu pharmacy company in Shanghai. *Paris polyphylla var* (3010030202), *yunnanensis*, *Gynostemma pentaphyllum* (702041), *Ophiopogon intermedius D.Don* (100224), and *Trigonella foenum-graecum L* (611066) purchased at Yueyang Hospital. They were mixed and crushed according to the weight ratio of 1.5:1:1:1. Then, five volumes of 70% alcohol and 30% pure water were added and the samples were extracted by ultra-sonication three times (60 min each time). The supernatant was collected and the alcohol was removed through rotary evaporation and then freeze dried it into powder. For *in vitro* experiments, the YYWY powder was dissolved in culture medium. The culture medium without YYWY was adopted as a control (**Figure S1**).

### Mouse Xenograft Assay

The animal experiments were approved by the Ethics Committee of Yueyang Hospital of Integrated Traditional Chinese and Western Medicine, Shanghai University of Traditional Chinese Medicine. The Lewis lung cancer cells were suspended in 200 μl PBS at 1×10^6^ cells/ml and injected into right flanks of 6-week-old C57BL/6 female mice. Mice were divided into three groups (n = 8): control group (0.9% normal saline/day for 30 days), YYWY group (18.8 g/kg), and DDP (cisplatin) group (2 mg/kg, once every 4 days). Tumor sizes were monitored by measuring the length (L) and width (W) with the help of calipers. Volumes were calculated using the formula (L × W^2^)/2.

### RNA-Seq Assay and Data Analysis

Based on the manufacturer's instructions, total RNA was isolated from tumor tissue using the Trizol reagent (Invitrogen). Samples with OD (260/280) ratios in the range of 1.8–2.0 and OD (260/230) ratios from 1.8 to 2.2, as identified through a NanoDrop Spectrophotometer, met the requirement of sequencing. RNA samples with RNA integrity numbers (RINs) greater than 7 and 28s/18s greater than 1.0 were selected for the subsequent RNA sequencing which was performed using an Agilent 2100 bioanalyzer. Also, 200 ng of total RNA was used to prepare the sequencing libraries by the application of Illumina TruSeq Stranded Total RNA Sample Preparation Kit according to the manufacturer's protocol. RNA sequencing was performed by BGI Genomics using BGISEQ-500 platform at Wuhan, China.

The high-quality sequencing reads were aligned to the mouse transcriptome (mm10, UCSC) using Burrows-Wheeler Aligner (BWA, v0.7.15a) (Kuo, 2008). The gene expression level was measured by fragments per kilobase of transcript per million fragments (FPKM). Fold change of FPKM≥2 and false discovery rate (FDR) cutoff value ≤ 0.001 were applied to evaluate differentially expressed genes (DEGs) with high levels of between-groups statistical significance. For enrichment analysis, Gene Ontology (GO) and Kyoto Encyclopedia of Genes and Genomes (KEGG) analyses were executed by enrichGO and enrichKEGG functions of clusterProfiler package, respectively (Li and Durbin, 2010) with the significance level of p.adjust (FDR) < 0.05.

### Cell Culture

Immature DCs were cultured from monocytes as described (Fan et al., 2015). DCs were generated from bone marrow (BM) cells obtained from 6- to 7-week-old male mice. In brief, BM cells were flushed from femurs and tibias. The culture of DCs started with a concentration of 1.0 × 10^6^ cells/ml in 12-well plates with RPMI-1640 (Gbico, NY. USA) supplemented with GM-CSF (315-03-20), rmIL-4 (214-14-20) (PeproTech, NJ, USA), 10% FBS (Gibco, NY, USA), 2 ml per well. Cells were cultured in a humidified chamber at 37°C and 5% CO_2_. After incubation for 24 h, the medium with non-adherent cells was replaced with fresh medium. The culture medium was removed and replenished with fresh medium every 2 days. The matured DCs were harvested for stimulation of following assays on the 7th day. The DCs were harvested and, following harvesting, the DCs were pulsed overnight with a Lewis cells lysate (1 × 10^5^ cells/well) to allow the DCs to capture and process the tumor-associated antigens for the next experiment co-cultivation. Mouse Lewis lung carcinoma (Lewis), human lung cancer cell lines H460 and human normal bronchial epithelial cells (16HBE) were obtained from cell bank of Chinese Academy of Sciences of Shanghai. Cells were cultured in RPMI 1640 medium supplemented with 10% FBS and 100 units per ml penicillin‐streptomycin solution at 37°C, 5% CO_2_ in a humidified incubator.

### Cell Viability Assay

Cell viability was estimated with the Cell Counting Kit-8 (CCK-8) assay kit (Dojindo, Kumamato, Japan). Absorbance was calculated for all samples at 450 nm (OD450). Cell viability rates were measured in 24 h and were calculated based on OD450 values. Cell viability rate (%) = A450(test)/A450 (control) × 100%.

### Cell Apoptosis Assay

Cells were harvested and washed after incubation for 24 h. Cell apoptosis was analyzed using an Annexin V-FITC/PI Apoptosis Detection Kit (V13241) (Thermo, MA, USA) per manufacturer's instructions. After staining, all samples were immediately measured on a CytExpert flow cytometer (CytoFLEX S, Beckman Coulter, USA). CytExpert Software (CytoFLEX S, Beckman Coulter, USA) was applied to analyze data.

### Phenotypic Characterization by Flow Cytometry Assay (FCA)

Phenotypic maturation of DCs was analyzed by flow cytometry, cells were resuspended in PBS containing 2% FBS and stained with APC-conjugated anti-CD11c antibody, PC7-conjugated anti-CD40, PC5.5-conjugated anti-MHCII, FITC-conjugated anti-CD83 and PE-conjugated anti-CD86 anti-body (Biolegend, CA, USA). Non-treated DCs were also stained with isotype IgG as negative control. To collect DCs and CD3 co-cultured cells, splenocytes from the same mouse strain were obtained, and incubated with anti-CD3 beads followed by magnetic sorting (MACS). DC at 2 × 10^4^ cells/well from the BM of C57BL/6J were seeded in 96-well round-bottom plates, pulsed with Lewis cell lysates (5 μg/ml) and incubated with YYWY (50 or 100 μg/ml), LPS or un-treated at 5μg/ml was added and incubated with cells for 24 h at 37°C. CD3 cells per well and co-cultured with DC (2 × 10^4^/well) in 96-well round-bottom plate for 96 h. Then, the surface markers were stained with FITC-conjugated anti-CD8 (Biolegend, CA, USA), before lysis of the cell membrane with BD Cytofix/Cytoperm™ ContentsFixation, then the surface markers were stained with FITC-conjugated anti-CD8 (Biolegend, CA, USA), before lysis of the cell membrane with BD.

### Real-Time Quantitative PCR Assay (qRT-PCR)

For cell experiments, total RNA was extracted using the Trizol (Sigma, CA, USA) method. cDNA was synthesized using the PrimeScript II 1st Strand cDNA Synthesis Kit (Takara, Tokyo, Japan). The expression of mRNA was measured by RT-PCR with SYBR Green PC Master Mix (Applied Biosystems, USA). Thermocycler conditions consisted of initial holds at 50°C for 2 min and 95°C for 5 min followed by a PCR program of 95°C for 15 s, 60°C for 15 s, and 72°C for 30 s for 40 cycles and a final hold at 72°C for 5 s. Reactions were executed by an ABI PRISM^®^ 7300 Sequence Detection System (Applied Biosystems, Canada). Data for all samples was normalized to the control. The expression of mRNA was calculated using the relative quantification equation (RQ = 2−^ΔΔ^Ct). The primer sets are shown in **Table 1**.

**Table 1:** Primers utilized for real-time PCR.

Gene	Forward	Reverse
**IL-12**	AGAGGTGGACTGGACTCC CGA	TTTGGTGCTTCACACTTCAG
**TNF-α**	GCGACGTGGAACTGGCAGAAG	GCCACAAGCAGGAATGAGAAGAGG
**IL-1β**	ATGGCAATGTTCCTGAACTCAACT	CAGGACAGGTATAGATTCTTTCCTTT-
**IFN-γ**	CCACAGCCCTCTCCATCAACTATAAGC	AGCTCTTCAACTGGAGAGCAGTTGAGG
**IL-2**	CCTCAACTCCTGCCACAATGT	TGCGACAAGTACAAGCGTCAGT
**β-Actin**	TGGAATCCTGTGGCATCCATGAAAC	TAAAACGCAGCTCAGTAACAGTCCG

### Western Blotting Analysis

Western blotting (WB) was conducted to evaluate the protein levels in cancer tissues and lung cancer cells. Protein concentrations were determined using the BCA Protein Assay Kit (Thermo, MA, USA). Proteins were detected using primary antibodies against (Cell Signaling Technology) p-JNK, JNK, pERK1/2, ERK1/2, p-p38, p38, TLR4, MyD88, IκBα, p65, β-actin, and (Abcam, Cambridge, MA) IKKα and IKKβ. Peroxidase-conjugated secondary antibody (Jackson ImmunoResearch Laboratories, USA) was used and immunoreactive bands were visualized based on the Enhanced Chemiluminescence (ECL) detection system (Thermo, MA, USA). The phosphorylation level of the selected protein was expressed as the ratio of the phosphorylated protein to total protein.

### Enzyme-Linked Immunosorbent Assay (ELISA)

Responder T cells used for allogenic T cell reaction were isolated with a MACS CD4^+^ isolation kit (Biolegend, CA, USA) from the whole spleen cells of C57BL/6 mice. DCs and CD4 co-culture supernatant are collected. Detection of TNF-α, IL-2, IFN-γ, IL-4, and IL-5, levels in the supernatants were determined using ELISA, according to the manufacturer's protocol (BD Biosciences, New Jersey, USA).

### Transmitted Electron Microscopy (TEM)

Samples were fixed in 2% paraformaldehyde and 2.5% glutaraldehyde in PBS at room temperatue for 1 h, then stored at 4°C until processing, followed by a secondary fixation in 1% aqueous osmium tetroxide with potassium ferricyanide overnight at 4°C. Then, samples were dehydrated in a graded series of ethanol, with propylene oxide acting as a transitional solvent and infiltrated in Epon. Sections were cut on a Lecia Uitracut Rultramicrotome in the range of 50- to 70-nm thickness and post stained with 5% uranyl acetate in 70% methanol and Sato's lead stain. Sections were viewed in a Tecnai G2 Spirit Bio TWIN.

### Immunofluorescent Staining

Paraffin-embedded specimens were sectioned at 4-μm thickness. The antigen retrieval was applied in a pressure cooker for 30min with the citrate buffer (pH 6.0), and they were blocked in PBS containing with 10% goat serum for 60 min at 37°C. After that, the sections were incubated with antibodies specific for rabbit-anti-mouse CD11c, or rabbit-anti-mouse CD8 (Bioss, Beijing, China) with an enveloping fluorescent probe overnight at 4°C. DAPI (Solarbio, Shanghai, China) was then used to counterstain the nuclei and images were obtained by fluorescence microscope.

### Hematoxylin-Eosin Staining

Hematoxylin and eosin (H&E) staining tissues from mice were embedded in paraffin blocks and subjected to H&E staining. Briefly speaking, sections were deparaffinized in xylene and rehydrated with in gradient ethanol (100%, 95%, 80%, and 70%). They were then stained with hematoxylin for 10 min, rinsed with 1% hydrochloric acid alcohol for 2 s, rinsed with tap water for 15 min, stained with eosin for 2 min, and rinsed again with distilled water.

### Immunofluorescence and Immunohistochemistry

According to the manufacturer's protocol, Ki-67, CD4, and CD8 protein expression in bladder cancer tissues and corresponding precancerous tissues were assessed by IHC with the 3'-diaminobenzidine (DAB) kit (Sigma, USA). Ki-67, CD4, and CD8 monoclonal antibody and goat anti-rabbit HRP antibody (Abcam, USA) were used.

### Statistical Analysis

Statistical analyses were performed by SPSS 24.0 (IBM Corp, Armonk, NY). The data are presented as the means ± SEM of three separate experiments. Statistical significance was determined using paired or unpaired Student's t-tests for standardized expression data.

## Results

### YYWY Exhibited Cytotoxicity Against Lung Cancer Cell Lines

**Figure 1:**
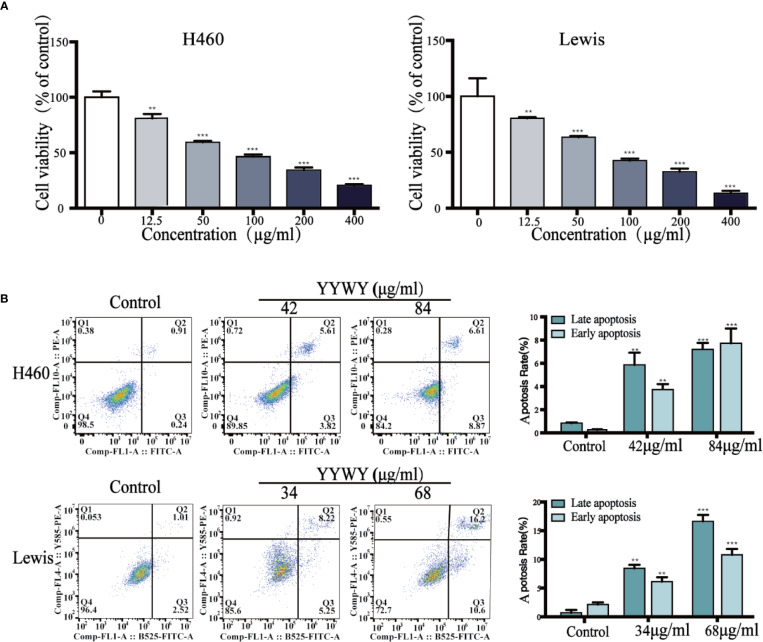
YYWY suppressed tumor cell growth in different cell line. **(A)** Cells were treated with various concentrations of YYWY for 24 h. The CCK8 assay was performed to assess cell viability. **(B)** YYWY induced apoptosis in H460 cells and Lewis cell. The cells were treated with 1/2IC50 and IC50 YYWY for 24 h. The percentage of apoptotic cells was measured by flow cytometry. The values represent the mean ± SEM. **p <  0.01, ***p < 0.001 compared to the control group.

We examined whether YYWY exerted cytotoxicity on human lung cancer cell lines NCI‐H460, mouse Lewis lung carcinoma cells, and Lewis and human normal bronchial epithelial cells (16HBE). The viability of H460 and Lewis cells treated with YYWY at different concentrations for 24 h was determined by CCK8 assay. As shown in **Figure 1A**, cell viability was decreased in these tested lung cancer cell lines in a dose-dependent manner when compared with 16HBE. Moreover, we calculated the half maximal inhibitory concentration (IC50) value based on cell viability. The IC50 of H460 and Lewis were 84.39 and 68.87 μg/ml, respectively.

In order to analyze the apoptotic features of YYWY‐treated lung cancer cells, we performed the Annexin V‐FITC/PI double staining assay. Compared with the control, the late apoptotic cells and early apoptotic cells were significantly increased in dose‐dependent manner upon YYWY treatment for 24 h in Lewis and NCI‐H460 cells (**Figure 1B**).

### YYWY Exerted Anti-Tumor Effect in Lewis-Bearing Mice

**Figure 2:**
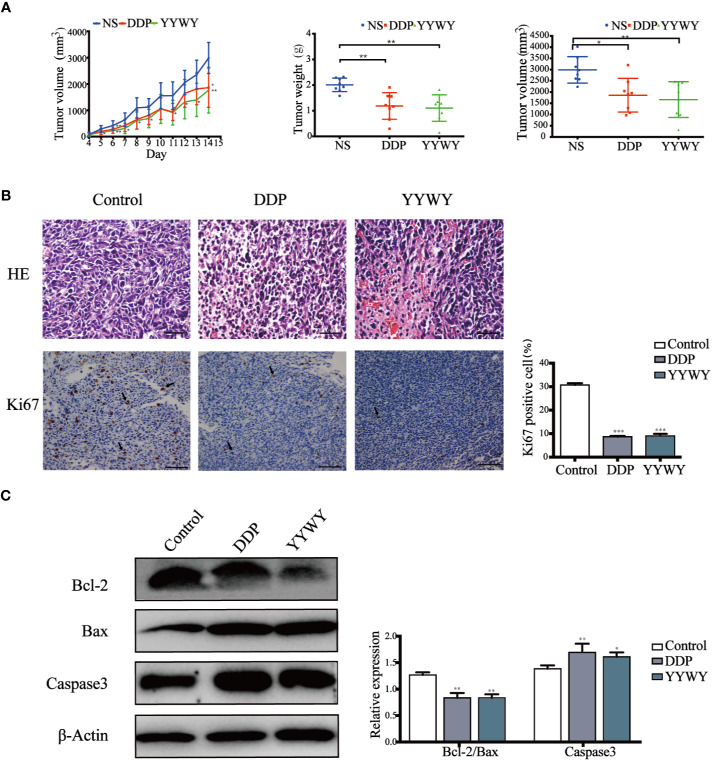
YYWY suppressed tumor growth in NSCLC-bearing mice. Lewis cells were subcutaneously implanted into C57BL/6 mice; and the model mice were administrated with 0.9% normal saline (200 ul, 14 days, ig.), YYWY (18.8 g/kg/day, 14 days, ig.) or DDP (2 mg/kg/4 days, 30 days, ip.) (n = 8), respectively. **(A)** Tumor volumes were calculated after measuring the length and width of the tumors daily using Vernier calipers every day. At the endpoint, the tumor tissues were harvested for the detection of the tumor weight and tumor volume. **(B)** Representative H&E and immunohistochemical staining of Ki67 were carried out in the tumor tissue from Lewis-bearing mice. Ki67 cells positive for the indicated proteins were counted from 5 fields for each tumor sample. Scale bar: 100μm. **(C)** Expression of Bcl-2, Bax and Caspase3 were measured by WB. β-Actin is a loading control. The values represent the mean ± SEM. *p < 0.05, **p < 0.01, ***p < 0.001 compared to the control group.

To evaluate whether YYWY inhibited the tumor growth *in vivo*, xenograft mouse models were established by subcutaneous injection of Lewis cells into the right flank of C57BL/6 mice. Daily treatment of YYWY at the concentration of 18.8 g/kg could significantly inhibited the tumor growth in Lewis-bearing C57BL/6 mice compared with the controls (**Figure 2A**). Furthermore, H&E stains, Ki67 and apoptosis assay were used to evaluate the effect of YYWY on the cell density, proliferation, and apoptosis of cancer cells *in vivo*. As shown in **Figure 2B**, compared with the control, the proliferation marker Ki67 immunostaining results showed a significant suppression of cell proliferation in the YYWY group mice; and a noticeable loose cell density and lymphocytic infiltration were found in pathological sections of the tumor xenografts in YYWY group (H&E stains). Moreover, the apoptosis was markedly increased within tumors of the YYWY group mice versus the controls, as evidenced by the up-regulation of caspase-3 expression and the Bax/Bcl2 ratio (**Figure 2C**).

### YYWY Enhanced the Immune Response in Lewis-Bearing Mice

**Figure 3:**
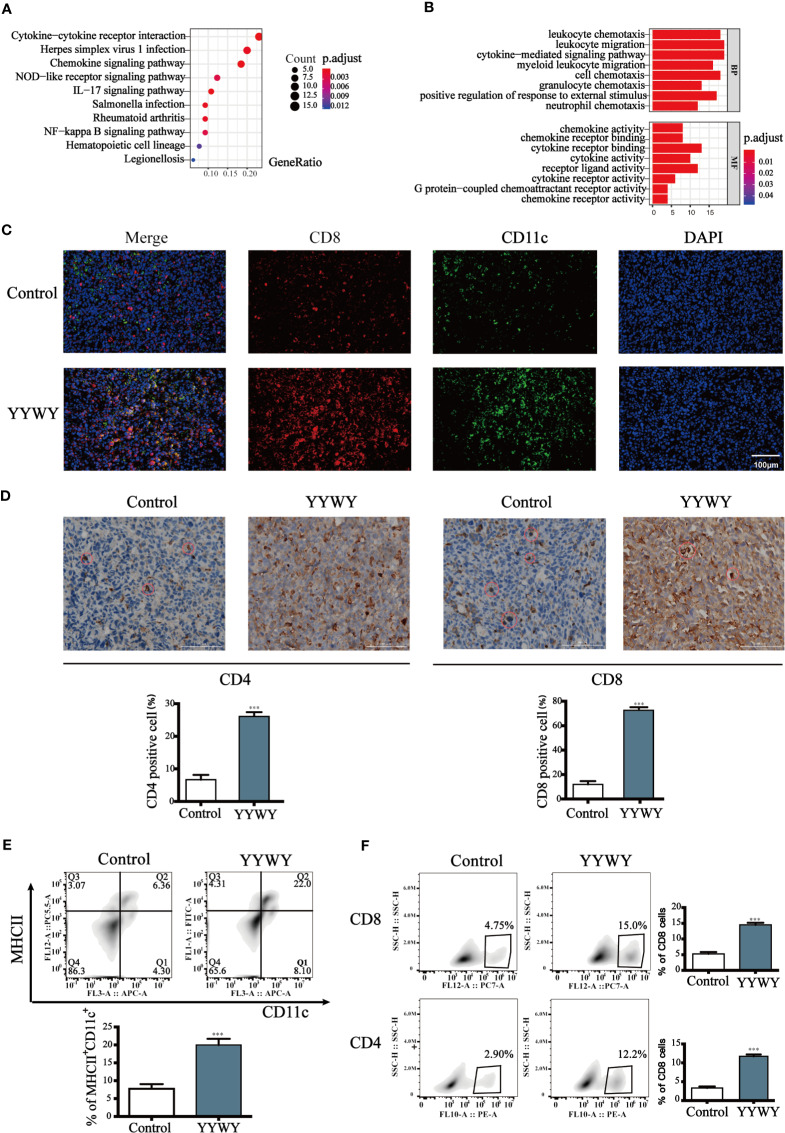
YYWY exerted anti-tumor effects through enhancing the immune response in Lewis-bearing mice. **(A, B)** KEGG pathway and GO enrichment analysis for the up-regulated genes of the tissue tumor in YYWY treated Lewis-bearing mice vs. controls (n = 3). **(C)** Immunofluorescence of the CD11c (red) and CD8 (green) combined with DAPI staining (blue) in tumor tissue from Lewis-bearing mice. Scale bar: 50 μm. **(D)** Representative immunohistochemical staining of CD4 and CD8 T cells were carried out in the tumor tissue from Lewis-bearing mice. Scale bar: 100μm. **(E)** Production of the DCs by spleen stimulated with YYWY or 0.9% normal saline (control) for 14 day as determined by flow cytometry. **(F)** Proliferation of CD4^+^ and CD8^+^ T cell in spleen stimulated with YYWY and 0.9% normal saline. The values represent the mean ± SEM of eight independent experiments. ***p < 0.001 compared to the control group. RNA-SEQ was performed in triplicate on the tumor tissues from Lewis-bearing mice and the controls.

The mRNA of the tumor tissues from Lewis-bearing model underwent RNA-seq. Subsequently, GO and KEGG enrichment analysis were conducted to provide clues of the pathways YYWY regulated. As shown in **Figure 2A**, the significant pathways for YYWY-related mRNAs were mainly enriched in the immune pathway, such as cytokine-cytokine receptor interaction and chemokine/NOD-like receptor/IL-17/NF-κB signaling pathways. Similarly, the most significantly enriched GO terms for YYWY also focused on the immune, including leukocytes chemotaxia, migration and chemokine/cytokines activity, as shown in **Figure 3B**.

Furthermore, the immunostaining results showed that YYWY increased the co-infiltration of CD11c^+^ DCs and CD8^+^ T cells (**Figure 3C**). Moreover, the number of CD4^+^ and CD8^+^ T cells in the tumor tissue of YYWY group both increased (**Figure 3D**), which may be caused by the increased number of tumor infiltrating DCs and T cells proliferation after YYWY treatment.

YYWY could promote the maturity of DCs as well as the responses of CD4^+^/CD8^+^ T cell in the tumor tissue. Therefore, we evaluated the DCs and CD4^+^/CD8^+^ T responses in splenocytes from mice. Antitumor immunity is initiated by APCs, such as DCs, which capture tumor antigens from tumor cells and induce the DCs and CD4/CD8 response in spleen. Afterwhile, we assessed whether YYWY could induce DC's CD4 and CD8^+^ T cell expression in tumor-bearing mice. The results showed that YYWY induced the expression significantly in DCs (**Figure 3E**), CD4^+^ T cells and CD8^+^ T cells (**Figure 3F**), compared with the control spleen group. These results indicated that YYWY could activate the immune response of controlling the growth of inoculated LLC cells.

### YYWY Promoted the Maturation of DCs

**Figure 4:**
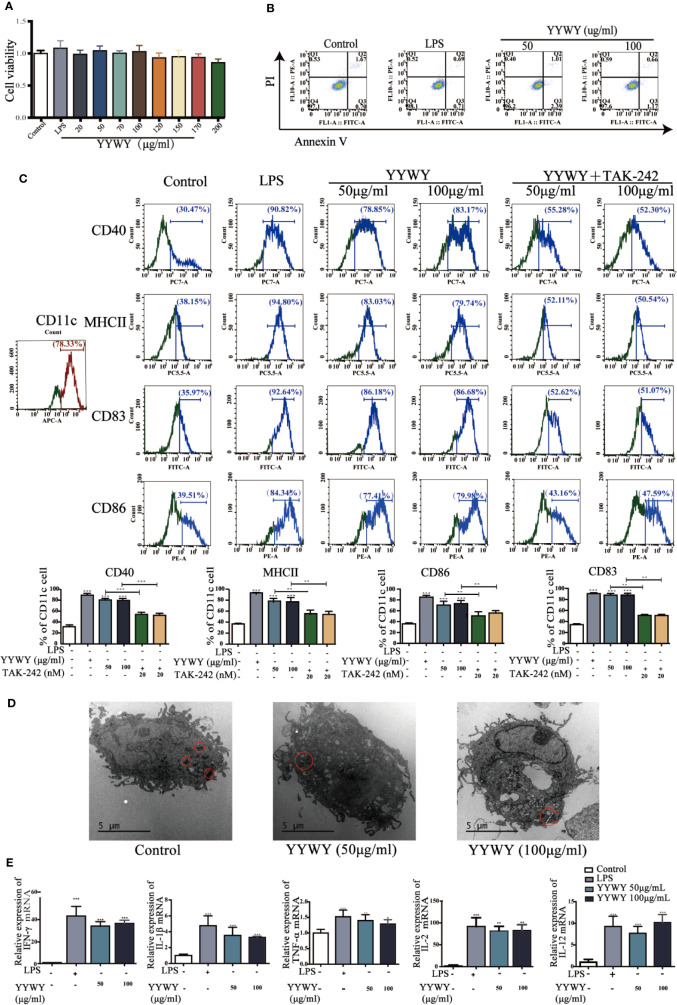
YYWY promoted the maturation of DCs. **(A)** BMDCs was treated with YYWY or LPS for 24 h. The cytotoxicity of YYWY on BMDCs was determined by CCK-8 assay. **(B)** Analysis of apoptosis by Annexin V/PI double-staining assay of BMDCs after YYWY (50 or 100 μg/ml) or LPS (1 μg/ml) treatment for 24 h, and the representative FACS analysis scatter grams of Annexin V-FITC/PI staining are presented. **(C)** BMDCs were treated with YYWY (50 or 100 μg/ml) for 24 h in the presence or absence of a pre-treatment of TLR4 blocker TAK-242 for 24 h, then DCs maturation markers (CD11c, MHCII, CD40/83/86) was detected by flow cytometry. Representative histograms displaying levels of fluorescent CD80, CD83, and CD11c are presented. The percentage of CD80^+^ and CD83^+^ cells in total CD11c^+^ cells are presented on the below. **(D)** The ultrastructure of BMDCs after YYWY treatment were observed through TEM. Scale bar: 5μM. **(E)** The mRNA levels of DCs-secreted cytokines (IFN-γ, IL-1β, TNF-α, IL-2, and IL-12) were detected *via* qPCR. The values represent the mean ± SEM of three independent experiments. *p < 0.05, **p < 0.01, ***p < 0.001 compared to the control group.

The critical role of immunity or immune system in the anti-tumor effects of YYWY calls for the exploration for the underlying mechanisms of YYWY in the immune. Since DCs are the initiator and modulator of the immune responses, we focused on the function of DCs in linking innate to adaptive immunity (Ardavín et al., 2004). To evaluate the effect of YYWY on the maturation of DCs, BMDCs were stimulated with LPS or YYWY for 24 h, then we analyzed the expression of DCs maturation markers and the secreted cytokines. First of all, based on the CCK-8 assay and Annexin V/PI detection, YYWY (50 and 100 μg/ml) did not induce obvious cell viability alterations and apoptosis compared with the controls. This result showed that the proliferation and apoptosis of DCs were not affected under the concentration of YYWY which were used to intervene BMDCs (**Figures 4A, B**). As shown in **Figures 4C, E**, YYWY alone indeed induced the increased number of CD11c^+^ CD40^+^/MHCII^+^/CD86^+^/CD83^+^ DCs as well as the expression of mRNA levels of IFN-γ, IL-1β, TNF-α, IFN-γ, IL-2, and IL-12. A further ultrastructure observation of DCs showed a significant proliferation of intracellular organelles, especially lysosomes after 100 μg/ml YYWY treatment (**Figure 4D**) (Zou et al., 2011). These findings were proved to be reasonable that YYWY could promote the maturation of BMDCs and the release of important proinflammatory cytokines, which could lead to a significant enhancement of their immune-stimulatory properties.

### YYWY Enhanced DCs-Induced Proliferation and Programming of T Cells

**Figure 5:**
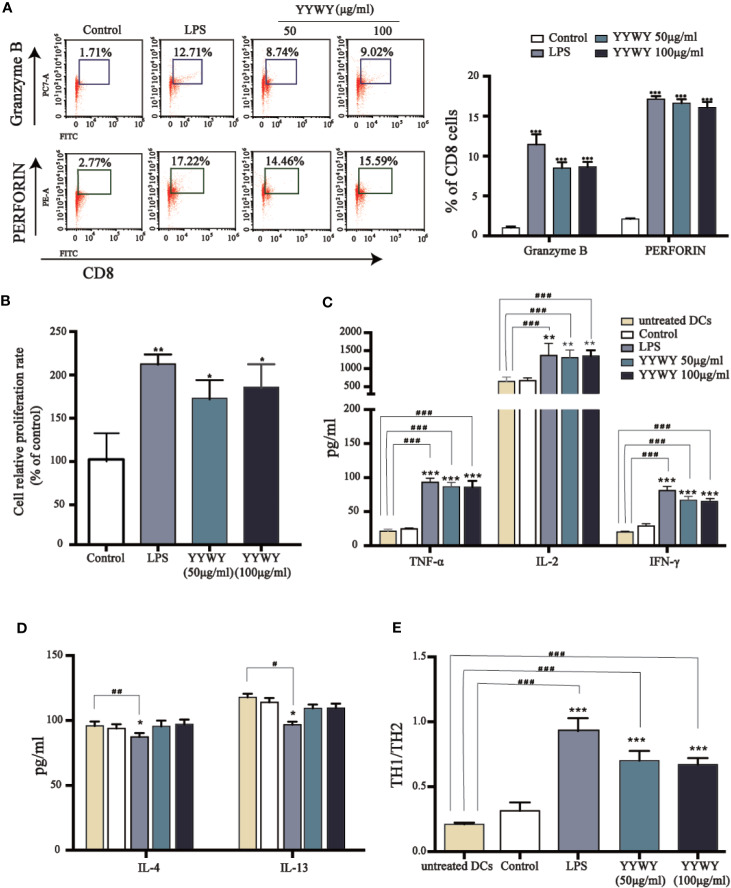
YYWY enhanced DCs-induced proliferation and programming of T cells. BMDCs were treated with YYWY (50 or 100 μg/ml) for 48 h, followed by collecting the DCs for subsequent co-culture with allogeneic CD3^+^ T cells for another 48 h. **(A)** The detection of PERFORIN and GRANZYME through flow cytometry was used to assess the differentiation of CD3^+^ T cells. Representative FACS analysis scatter grams are presented on the left. The quantification of PERFORIN and GRANZYME-positive cells which were normalized to total CD3^+^ T cells is presented on the right. **(B)** The proliferation of CD4^+^ T cells was estimated by CCK-8 assay. **(C–E)** The cytokine levels of Th1 type cytokines (IL-2, TNF-α, IFN-γ), Th2 type cytokines (IL-4, IL-13), and Th1/Th2 (IFN-γ/IL-4) radio were detected by Elisa assay. The values represent the mean ± SEM of three independent experiments. *p < 0.05, **p < 0.01, ***p < 0.001 compared to the control group, ^#^*p* < 0.05, ^##^*p* < 0.01, ^###^*p* < 0.001 compared to the untreated DC.

The main function of DCs is to integrate the infection or damage signals and present processed antigen to naive T cells and then assist to guide T cell differentiation. Therefore, we investigated the T cell proliferation and priming potential of murine BMDCs in the presence of YYWY. YYWY (50 or 100 μg/ml) pre-treated BMDCs for 24 h, followed by collecting DCs for subsequent co-culture with allogeneic CD3^+^ primary T cells for another 96 h. YYWY-treated DCs significantly stimulated CD3^+^ T cells to differentiate into CD8^+^CTL cells, which was based on the PERFORIN and GRANZYME detection *via* flow cytometry (**Figure 5A**). According to the previous method, YYWY-treated DCs and CD4^+^ T were co-cultured. The results showed that YYWY could induce the proliferation of CD4^+^ T cells (**Figure 5B**) and increase the secretion of Th1-type cytokines (TNF-α, IL-2, and FN-γ), which indicated that YYWY-treated DCs preferred to induce CD3^+^ T differentiation into Th1 (**Figure 5C**). While co-cultivation of YYWY-treated and CD3^+^ T cells had no significant change on Th2 lineage development (**Figure 5D**). In addition, DCs treated with YYWY could promote the ratio of Th1/Th2 (IFN-γ/IL-4 radio) (**Figure 5E**).

### YYWY Activated MAPK and NF-κB Signaling Pathways in DCs

**Figure 6:**
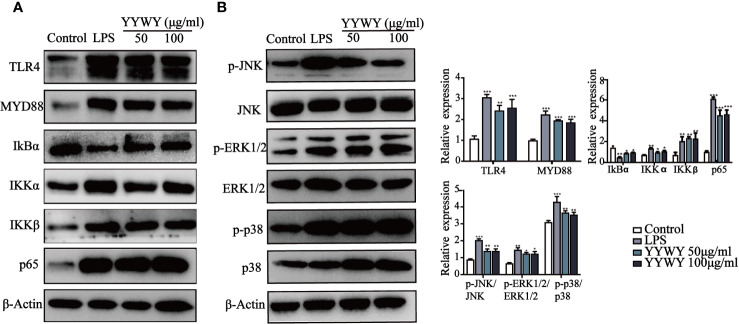
YYWY activated MAPK and NF-κB signaling pathways in DCs. The expression of **(A)** NF-κB (TLR4, MyD88, IKKα/β, IκBα, and p65) and **(B)** MAPKs (JNK, JNK, p-ERK1/2, ERK1/2, p-p38 and p38) were examined by western blotting. The values represent the mean ± SEM of three independent experiments. *p < 0.05, **p < 0.01, ***p < 0.001 compared to the control group.

Since previous studies have illustrated that MAPK and NF-κB signaling pathways played critical roles in the maturation of DCs (Minadakis et al., 2019). We further explored its role in YYWY-induced DC maturation. **Figure 2C** showed that TLR4 blocker TAK-242 could partially reverse the increased number of CD11c^+^ CD83^+^/86^+^ DCs induced by YYWY, which pointed to the importance of TLR4 signaling cascade in the effect of YYWY. Moreover, treatment of YYWY on BMDCs significantly increased the levels of TLR4, MyD88, IKB-α, IKKα/β, and NF-κB (p65) as well as decreased the expression of IκBα. These results implied an activated TLR4-NF-κB pathway (**Figure 6A**).

As shown in **Figure 6B**, YYWY promoted the phosphorylation of JNK, ERK1/2, and p38, which resulted from the activation of JNK, ERK1/2, and p38 signaling pathways after the treatment of YYWY. All these data suggested that YYWY regulated both MAPK and NF-κB signaling pathways to augment the immune-stimulatory capacity of DCs.

## Discussion

The pathology of NSCLC is complex and largely unknown, which greatly increase the difficulty in the treatment of NSCLC. Available chemotherapeutics NSCLC have various limitations such as drug tolerance, unsatisfied outcome, and high price. Therefore, novel targeted therapeutic drugs are needed. Nowadays, TCM has been recognized as an alternative approach for cancer therapeutics. Increasing evidence revealed TCM reduced the toxicity of chemotherapy and radiotherapy as well as alleviated the symptoms of cancer. Importantly, numerous studies (Xu et al., 2013; Liu Y.-X. et al., 2019; He et al., 2020) have reported many TCM could increase survival rates of cancer patients in the clinical and preclinical studies. In this study, we revealed that the immunoregulatory activity of YYWY formula played a critical role in its anti-NSCLC effects. YYWY effectively promoted the maturation of DCs, which enhanced the proliferation and differentiation of T cells into Th1 and CTL through MAPKs and NF-κB signaling pathways.

YYWY formula was rationally developed from the JFK formula which was approved by the China Food and Drug Administration (CFDA) for the treatment of NSCLC (NO.z 19991043). The simplified formula YYWY is consists of *Paris polyphylla*, *Gynostemma pentaphyllum*, *Liriope graminifolia*, and *Fenugreek*. In the present study, we investigated the anti-tumor activity of YYWY and explored the underlying mechanisms in our study. YYWY inhibited cell proliferation and promoted the apoptosis of lung cancer cells (**Figures 1A, B**). Administration of YYWY (18.8 g/kg) significantly suppressed the the tumor growth in Lewis-bearing C57BL/6 mice (**Figure 2A**). These data confirmed the anti-lung cancer effect of YYWY *in vitro* and *in vivo*.

Meanwhile, a non-discriminatory RNA-seq analysis and the corresponding enrichment analysis confirmed the immune system, including the elevated cytokine-cytokine receptor interaction, chemokine, and cytokine-mediated signaling pathways and cytokine receptor binding, may be the outstanding mechanism in the anti-tumor effect of YYWY (Aguilera-Aguirre et al., 2015) (**Figures 3A, B**). We performed a preliminary investigation of immune cells, and the results showed a significant infiltration of DCs, CD4^+^ and CD8^+^ T cells in the tumor tissue from YYWY-treated Lewis-bearing mice. DCs activation plays an important role in the formation of tumor immunopromotive microenvironment. DCs present antigenic peptides to CD4^+^ and CD8^+^ T cells *via* MHC-II and MHC-I, respectively (Mulders et al., 1999; Jing et al., 2020).

Moreover, among the YYWY-responsive KEGG and GO, the significant enrichment processes which included of chemokines, cytokines, integrin, and interleukin signaling pathways had a closed relationship with the activation of DCs. DCs function is a bridge between the innate and adaptive immune systems and acts as attractive targets for tumor immunotherapy because of their unique ability of activating primary immune responses through the presentation of antigens to naïve CD4^+^ and CD8^+^ T cells (Li et al., 2017; Evelyn et al., 2019; Nobis et al., 2019). The nature of DCs in the subsequent immune response is partially determined by the maturation status of DCs (Kapsenberg, 2003). Matured DCs improve the motility and maximize surface area for T cell interactions by up-regulating surface expression of MHC and costimulatory molecules (CD86/83) and undergoing cytoskeletal changes (Sansom et al., 2003; Blanco et al., 2008). Maturation also induces DCs to secrete cytokines and chemokines to promote the proliferation and differentiation of naïve T cells (Karolina and Jacques, 2012; Janostiak et al., 2017; Liu W.-L. et al., 2019). In this study, we firstly paid attention on the initiation factor and linking bridge in the immune system, DCs. Treatment of YYWY induced high expression of co-stimulators and increased secretion of cytokines (**Figures 4C, E**), suggesting that YYWY strongly induced the maturation of DCs.

It has been reported that T cells and their associated cytokines are abnormal in cancer patients with cancer and are involved in tumor development and progression. The antitumor responses mediated by CD8^+^ T and Th1 cells was suppressed in patients with NSCLC (Song et al., 2019). Our data further reported YYWY-matured DCs promoted the proliferation and the differentiation of primary T cells into Th1 cells and CTLs (**Figures 5A, C**), increasing Th1/Th2 radio (**Figure 5E**). These results were consistent with the previous findings (Athanasios et al., 2016; Xinyu et al., 2018). It has been reported that activated DCs released IL-2, IL-12, IFN-γ, and TNF-α, which prompted CD4^+^ Th1 differentiation and accelerated Th1-mediated antitumor responses (Dranoff, 2004). Th1 cells secreted pro-inflammatory cytokines in order to activate downstream effector responses and support CD8^+^CTL maturation, forming the basis for antitumor immunity. The polarization of T cell, especially differentiation into CD4^+^ Th1 and CD8^+^CTL cells, is of particular interest to cancer immunotherapy (Bedoui et al., 2016). Therefore, the immunotherapy of YYWY was demonstrated to be mediated by promoting the differentiation of T cells *via* maturation of DCs.

The key role of DCs in the anti-tumor effect of YYWY urges for a further exploration of the signaling pathways involved in its maturation. Numerous studies have reported mitogen-activated protein kinase (MAPK) and NF-κB signaling pathways were responsible for regulating the activation and maturation of DCs (Giovanelli et al., 2019). p38 and JNK activation generally caused maturation of DCs and secretion of pro-inflammatory cytokines, such as IL-12, TNF-α, IL-1β, IL-6, and IL-2 (Bedoui et al., 2016). Importantly, p38 activation up-regulated DCs co-stimulatory molecules and maturation markers such as CD86, CD83, and CD40 and MHC class II (De Benedetti et al., 2001; Nakahara et al., 2004; Escors et al., 2008). Signaling through NF-κB also determines the increased expression of MHC II and co-stimulatory molecules, release of pro-inflammatory cytokines and chemokines, and DCs recruitment to secondary lymphoid organs (Andreakos et al., 2006; Rath et al., 2006). In this study, the MAPKs and MyD88-NFκB pathways were significantly activated in DCs by YYWY treatment, indicating both of them were involved in the process of YYWY-matured DCs (**Figures 6A, B**). TLR interactions trigger the expression of proinflammatory cytokines as well as the functional maturation of antigen presenting cells of the innate immune system. Moreover, TLR4 plays an initiated role in both MAPKs and NF-κB signaling pathways (Reddy et al., 2006). That activating TLR trigger the expression of proinflammatory cytokines as well as the functional maturation of antigen presenting cells of the innate immune system. In our study, the blockade of YYWY-induced expression of co-stimulatory molecules of DCs by TLR4 inhibitor TAK-242 further suggested the importance of MAPK and NF-κB in the DCs maturation (**Figure 4C**).

## Conclusion

The present study reported the anti-lung cancer effects of YYWY *in vitro* and *in vivo*, and revealed the underlying mechanisms in which YYWY effectively promoted the maturation of DCs and enhanced the proliferation and differentiation of T cells into Th1 and CTLvia MAPKs and NF-κB signaling pathways.

## Data Availability Statement

The data generated for this study can be found in NCBI, using the accession number PRJNA587610.

## Ethics Statement

The animal study was reviewed and approved by Yueyang Hospital of Integrated Traditional Chinese and Western Medicine (No. 18806).

## Author Contributions

XZ, LX and BZ conceived and designed the study. BZ, XH, LJ, LW, LB, YY, WY, YY, SJ, and PH performed the experiments. CW provided the mutants. BZ and LJ wrote the paper. XZ reviewed and edited the manuscript. All authors read and approved the manuscript.

## Funding

This work is supported by National Natural Science Foundation of China (81704035, 81973810, 81904163), Action Plan of Shanghai Chinese Medicine for Three Years [ZY(2018-2020)-CCCX-2004-09], Chinese Medicine Association (QNRC2-C15), and Chinese Traditional Medicine Association Young Talents Lifting Project and Shanghai Science and Technology Commission “Sailing Plan”(19YF1450000).

## Conflict of Interest

The authors declare that the research was conducted in the absence of any commercial or financial relationships that could be construed as a potential conflict of interest.
