# Supplementary material for: A TCM Formula YYWY Inhibits Tumor Growth in Non-Small Cell Lung Cancer and Enhances Immune-Response Through Facilitating the Maturation of Dendritic Cells
Source: Front Pharmacol. 2020 Jun 9;11:798. doi: 10.3389/fphar.2020.00798 (PMC7301756; doi:10.3389/fphar.2020.00798)
Supplement: Supplementary file 1 [file DataSheet_1.docx]

**Supplementary Material HPLC**

**Sample preparation**

100 μL YYWY sample was mixed with 1.4 ml methanol. After vertexing for 3 min, the mixture was centrifuged at 13,000 rpm for 10 min. The supernatant layer was transferred into another tube and evaporated to dryness at 40 °C under nitrogen. The residue was reconstituted with 100 μL of mobile phase and 10 μL was injected into LC system for analysis.


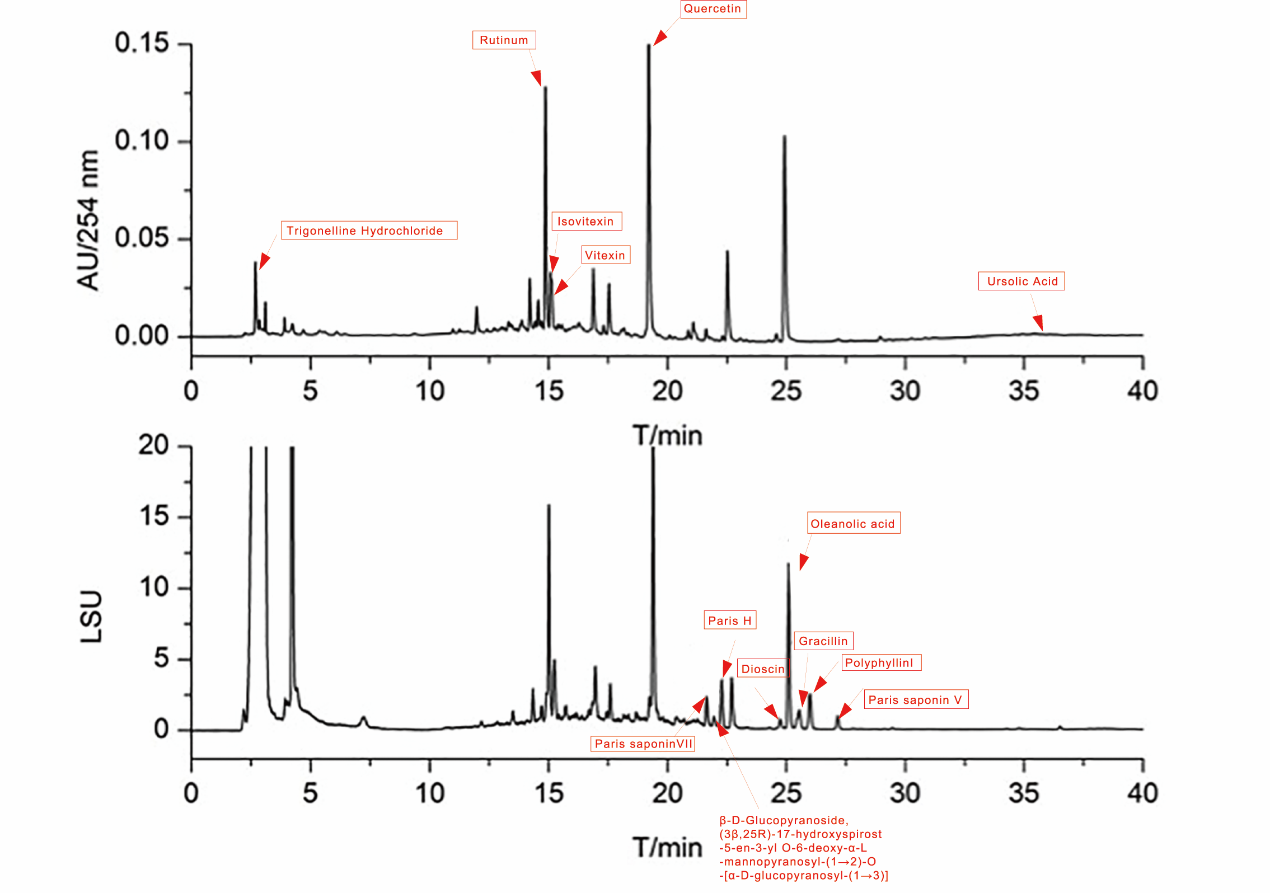


**SUPPLEMENTARY FIGURE 1 |** Representative chromatographic profile of YYWY The constituent of YYWY was assessed by HPLC on Agilent HPLC 1200 system (Agilent, USA). The chromatographic separation was achieved using Unitary C18 (4.6 × 250 mm, 5 μm, Agilent, USA). The column temperature was maintained at 40 °C. The mobile phase consisted of 0.01% trifluoroacetate (TFA) water and acetonitrile (ACN). Flow rate was 1 mL/min and ultraviolet (UV) detection wavelength was 254 nm.
